# Research Progress of Polymer Biomaterials as Scaffolds for Corneal Endothelium Tissue Engineering

**DOI:** 10.3390/nano13131976

**Published:** 2023-06-29

**Authors:** Xiaoying Luo, Xin He, Hui Zhao, Jun Ma, Jie Tao, Songjiao Zhao, Yan Yan, Yao Li, Shenmin Zhu

**Affiliations:** 1 State Key Laboratory of Metal Matrix Composite, School of Materials Science and Engineering, Shanghai Jiao Tong University, Shanghai 200240, China; luoxy2001@sjtu.edu.cn (X.L.); jssyhx1@sjtu.edu.cn (X.H.); liyaosjtu@sjtu.edu.cn (Y.L.); 2 National Clinical Research Center for Eye Diseases, Shanghai Key Laboratory of Ocular Fundus Diseases, Shanghai Engineering Center for Visual Science and Photomedicine, Shanghai Engineering Center for Precise Diagnosis and Treatment of Eye Diseases, Shanghai General Hospital (Shanghai First People’s Hospital), Shanghai 200080, China; taojie123@sjtu.edu.cn (J.T.); zhaosj93@163.com (S.Z.); sjtu_yanyan@163.com (Y.Y.); 3 UniSA STEM and Future Industries Institute, University of South Australia, Mawson Lakes, SA 5095, Australia; jun.ma@unisa.edu.au

**Keywords:** biomaterials, corneal endothelium, tissue engineering, topography

## Abstract

Nowadays, treating corneal diseases arising from injury to the corneal endothelium necessitates donor tissue, but these corneas are extremely scarce. As a result, researchers are dedicating significant efforts to exploring alternative approaches that do not rely on donor tissues. Among these, creating a tissue-engineered scaffold on which corneal endothelial cells can be transplanted holds particular fascination. Numerous functional materials, encompassing natural, semi-synthetic, and synthetic polymers, have already been studied in this regard. In this review, we present a comprehensive overview of recent advancements in using polymer biomaterials as scaffolds for corneal endothelium tissue engineering. Initially, we analyze and present the key properties necessary for an effective corneal endothelial implant utilizing polymer biomaterials. Subsequently, we focus on various emerging biomaterials as scaffolds for corneal endothelium tissue engineering. We discuss their modifications (including natural and synthetic composites) and analyze the effect of micro- and nano-topological morphology on corneal endothelial scaffolds. Lastly, we highlight the challenges and prospects of these materials in corneal endothelium tissue engineering.

## 1. Introduction

**Figure 1:**
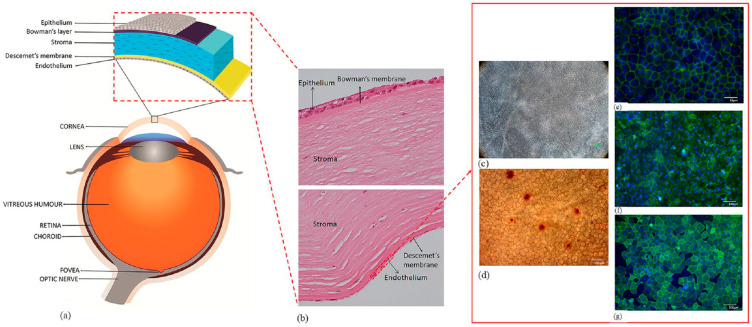
Anatomy of the human eye and cornea: (**a**) The figure illustrates different parts of the eye and layers of the corneal tissue; (**b**) the histological section of human cornea showing different layers of the tissue; (**c**) corneal endothelial cell viability using trypan blue staining observed under a light microscope; and (**d**) alizarin red staining showing the hexagonality of endothelial cells. Biomarkers, such as (**e**) ZO-1, (**f**) Tag-1A3, and (**g**) Tag-2A12, are utilized to determine the presence of specific proteins such as tight junctions and surface epitopes. These biomarkers are used on cultured corneal endothelial cells to characterize the cell type and for functional analysis [3]. Reprinted with permission from Ref. [3]. Copyright 2021, Sage Journals.

Incoming light is transmitted and focused onto the retina via the cornea, which is located at the front of the eye and has no blood vessels and good transparency. A cornea consists of five distinct layers, as schematically shown in Figure 1, with the corneal endothelium (CE) positioned as the posterior layer. The CE plays a crucial role in maintaining corneal transparency by regulating the dynamic hydration of the cornea, which is achieved through a delicate balance between a “leaky” barrier and active ionic pumps located on the corneal endothelial cells (CECs). Injury or dysfunction of these cells may result in impaired ion and solute transport, thereby causing corneal edema. The primary difference between the corneal endothelium and other bodily tissues or cells is the fact that CECs lack the ability to regenerate in vivo [1]. Therefore, it is crucial to maintain their health throughout life to prevent corneal edema.

Over 10 million individuals around the world are said to be affected by corneal disorders, which are the fifth most common cause of blindness. Currently, corneal transplantation is thought to be the best course of treatment for these conditions and is one of the most commonly performed transplant procedures worldwide. While endothelial dysfunction is the primary reason for corneal transplantation, corneal dystrophies are the primary cause of this dysfunction. Fuchs’ endothelial corneal dystrophy is the most prevalent corneal dystrophy, which contributes to around 39% of all corneal transplantations performed globally [2].

Penetrating keratoplasty and endothelial keratoplasty (EK) are the two primary kinds of corneal transplantation. Penetrating keratoplasty entails replacing all layers of the recipient’s cornea with a donor cornea, while EK selectively replaces only the defective posterior layer of the cornea [4]. According to the 2021 Eye Bank Association of America report, more than sixty percent of all corneal transplants were EKs, making it the most popular keratoplasty [2].

One major predicament pertaining to keratoplasty is the restricted accessibility of appropriate donor tissue. It has been statistically established that the quantity of patients necessitating medical intervention is significantly greater than that of available donors [2]. Additionally, an obstacle associated with cadaveric donor transplantation is the prolonged risk of rejection and malfunction of an allogenic graft [5]. Considering that the endothelium is the most impaired and delicate tissue, it is imperative to explore additional possibilities, such as tissue engineering methods in vitro for corneal endothelium regeneration. Current options comprise cell therapy or combining cells with artificial substrates. Researchers can extract CECs from donor corneas by enzymatic digestion and achieve in vitro expansion of CECs using a medium that promotes cell expansion in vitro (e.g., medium containing specific growth factors) to activate signaling pathways (e.g., phosphatidylinositol 3-kinase (PI3K)/Akt and Smad2 signaling pathways), resulting in the proliferation of CECs [6]. Once these CECs are generated, they must either aggregate with one another to create a transplantable cell sheet or be combined with extracellular matrix (ECM) components, allowing later implantation. In the latter scenario, these cells must directly adhere to a substrate akin to Descemet’s membrane (DM). Biomaterials that can enhance cell function may be developed to improve the durability of transplanted grafts and the proliferation of CECs in vitro.

Even though there are some reviews published on various materials for corneal endothelial tissue engineering, the classification and generalization of material modifications need to be summarized. This paper aims to summarize the most recent developments in polymer-based biomaterials for corneal endothelium tissue engineering. The crucial characteristics of the corneal endothelial implant are outlined. Compared to other papers on similar topics, this article focuses on modifying the matrix based on a generalized analysis of natural and synthetic materials used for corneal endothelial scaffolds. The modifications are classified into composition design and topography regulation. The modification in the composition includes combining nanomaterials (e.g., nanofibers, carbon quantum dots, gold nanoparticles, and iron oxide nanoparticles). Furthermore, the influence of the topological morphology on the functionality of a corneal endothelial scaffold is analyzed, especially the nano-topography, including the design of nanopatterns.

## 2. Required Properties of the Corneal Endothelial Implants

Descemet’s membrane (DM) is an extracellular matrix situated posteriorly to the cornea and secreted by the corneal endothelial cells. Its main components include laminin, fibronectin, type IV and type VIII collagens, and perlecan. DM functions as a basal lamina for the corneal endothelial cells in a human cornea. To enhance the in vitro proliferation of corneal endothelial cells, promote the successful implantation of the scaffold, and optimize its endothelial functional capacity, it is imperative to ensure that the implants exhibit performance characteristics as similar as possible to those of DM. The basic properties include transparency, permeability, mechanical stiffness, topography, biocompatibility, degradability, etc.

The primary characteristic is transparency, which pertains to the corneal endothelium’s capacity to transmit light. DM exhibits a high level of transparency, reaching up to 90% within the visual range. The natural cornea’s refractive index is 1.367, which is critical to the refractive power of the eye. Refractive errors could occur even if its contour is slightly changed [7]. The implant must possess a refractive index that is as similar as possible to the corneal tissue and an adequate endothelial cell density to preserve the cornea’s normal light transmission (≥90%).

Subsequently, permeability should be taken into account. Permeability pertains to the capability of allowing nutritional elements to flow through the CECs. Aqueous humor provides most of the nutrients the cornea and the water need to preserve their typical hydrated states. Despite the fact that the cornea lacks blood vessels, the endothelium establishes a route for water and nutrients to receive from the anterior chamber to the cornea. Consequently, corneal endothelial implants must be permeable to biomolecules providing nutrients such as glucose and albumin. Generally, molecules with smaller molar masses exhibit greater permeability than those with larger molar masses. Glucose is a low-molecular-weight compound (0.18 kDa) and is considered the primary source of energy transfer in the cornea. It is present in the aqueous humor, penetrates through the endothelium into the corneal stroma, and metabolizes to produce lactic acid that subsequently diffuses back into the anterior chamber. The scaffold materials’ permeability can be evaluated by using the diffusion coefficient of glucose through the graft. As there is limited data in the literature on the permeability of DM, the corneal diffusion coefficient of glucose can be used as a reference value (3.0 ± 0.2 × 10^−6^ cm^2^ s^−1^).

Mechanical properties are indeed essential for corneal endothelial implants. Specifically, mechanical stiffness refers to the ability of an implant to maintain its shape and resist deformation. This property can be expressed in terms of tensile strength, Young’s modulus, elongation properties, and other similar measures. Since an implant serves as the support for the CECs, it must be able to withstand mechanical stretching and folding during implantation through small incisions in the eye while still retaining its shape and mechanical, physical, and optical properties once it is unfolded in situ. In addition, the implant should possess adequate viscoelasticity, which enables it to adapt to the changing curvature of the cornea during the healing process from its edematous state to the post-edematous state. The stiffness of an implant can significantly impact the behavior of the cells that adhere to it. Therefore, the mechanical strength of an implant should be similar to that of DM in order to provide an environment conducive to the expansion of natural corneal endothelial cells. Previous research has indicated that DM exhibits a tensile strength of ~2.6 MPa and Young’s modulus of ~50 KPa [8]. The thickness of the scaffold implanted by DMEK is 10–20 μm [9]. It is a daunting challenge to maintain mechanical resilience with such thickness. 

The substrate of an implant should ideally provide a suitable microenvironment that can support the viability, proliferation, and signaling pathways of CECs. To achieve this, the matrix used for culturing CECs should replicate the molecular, physiological, and mechanical properties of DM. This promotes cell expansion, cell adhesion, the deposition of the extracellular matrix, and the interactions between the cell layer and the scaffold. Topography is a key physical factor that can guide stem cells’ fate by providing surfaces with specific roughness that can influence the behavior of the attached cells [10]. The necessity of subtle surface roughness to enhance cellular behavior is attributed to the amplification of protein adsorption onto the substrate’s surface, which inherently boosts the CECs’ interaction with the substrate [11]. Various morphologies are fabricated through techniques such as replica molding or stereolithography [12]. In recent work, 3D confocal microscopy was employed to analyze the microtopography of DM [13]. The study revealed irregularly shaped, flat hexagonal pits that gave CECs a distinctive polygonal or hexagonal appearance. The pits had a width ranging from 10 to 20 μm and were up to 1 μm deep. According to the findings, CEC functionality was improved by replicating the native DM structure. Earlier research found that the growth of CECs varied with topographic features. When the topographic height and density of the substrate surface were low, CECs tended to form continuous monolayers more often. Still, this ability was diminished on substrates that were tightly packed. As a result, topographical cues might be employed to forecast CECs’ capacity to construct the required continuous monolayer [14]. Furthermore, achieving an optimal balance between hydrophilicity and hydrophobicity is crucial for promoting cell-surface interactions. Surface wettability and the properties of functional groups, such as carboxylic acid and amine groups, are also crucial factors that significantly influence cell adherence and modify the microenvironment for CECs [15].

Corneal endothelial implants should maintain continuous attachment to the posterior surface of the cornea. Research suggests that the hydrophilic external surface of the implant can support adherence to the cornea, eliminating the need for sutures or an additional binding agent [16]. During the healing process, the long-term adherence of an implant to corneal tissue is facilitated by bio-active attachment through protein adsorption, primarily fibronectin and laminin. Furthermore, the clever design of an implant shape, including the center and skirt, can also enhance its adhesion to the cornea.

The hydrophilic/hydrophobic nature of the materials is also important for corneal endothelial tissue engineering scaffolds. Hydrophilic materials commonly include gelatin, chitosan, polyethylene glycol (PEG), collagen, etc. [15,17,18]. Apart from promoting the adhesion of transplants to the posterior cornea, hydrophilic materials can help with the penetration of nutrients such as glucose and the adsorption of proteins. They have a significant role in promoting cell adhesion because of their high water absorption capacity. Hu et al. [18] reported the fabrication of gelatin/polycaprolactone (PCL) and collagen/PCL nanofiber scaffolds, demonstrating expected hydrophilicity, wettability, and biocompatibility. Bone marrow endothelial progenitor cells (BEPC) adhered well to collagen/PCL and gelatin/PCL scaffolds. On the other hand, hydrophobic materials used for corneal endothelial scaffolds include polycaprolactone (PCL), poly(D,L-lactic acid) (PDLLA), etc. [19,20]. Due to their hydrophobicity, nanofibrous membranes made from PDLLA have relatively low glucose permeability [19]. Additionally, the hydrophobicity of PCL makes it have a slow degradation rate, which plays a crucial role in modulating the degradation properties and mechanical properties of chitosan nanoparticle/PCL composites [20].

Both biodegradable and non-biodegradable materials can be utilized for corneal endothelial grafts. If the graft is designed to be permanently present in the body, then a non-degradable scaffold material, such as polymethylmethacrylate (PMMA), can be employed. Kruse et al. [19] reported using an electro-spun PMMA scaffold as a carrier for CECs, which exhibited cytotoxicity. The long-term safety and inflammatory response of non-degradable scaffolds in vivo may be of concern. As the CECs continue to secrete an extracellular matrix, the thickness of the DM increases over time, which is expected to replace the scaffold. Therefore, biodegradable materials are often studied as part of corneal endothelial tissue engineering scaffolds. If a material is biodegradable, its degradation rate must be synchronized with the rate of DM regeneration to avoid any adverse effects on the rest of the eye. Controlled biodegradation of the tissue-engineered matrix without producing toxic byproducts is crucial to restoring the natural structure, morphology, and function of the target tissue. The implanted materials and their breakdown products must be easily reproducible and have non-toxic qualities. Most natural materials, such as gelatin and chitosan, are degradable in vivo. At the same time, the most commonly used synthetic material is polycaprolactone, which has a relatively slow degradation rate. Tayebi et al. [20] constructed a biodegradable transparent scaffold for culturing corneal endothelial cells by incorporating chitosan nanoparticles into chitosan/polycaprolactone (PCL) membranes. The in vitro degradation of the chitosan nanoparticles/chitosan/PCL composite scaffold was tested by immersing the scaffold fragments in PBS (pH = 7.4) at 37 °C. The slope of the degradation rate of the scaffold was approximately constant over 21 days, and ultimately, approximately 76% of the scaffold degraded. The shape of the scaffold was almost preserved throughout the degradation assessment. Song et al. [21] combined poly(lactide-co-caprolactone) with an extracellular matrix to construct a transparent, biodegradable adhesion carrier for CECs. It is known that the long-term biodegradation profile in vivo is a key issue for transplanting biodegradable polymers or scaffolds in vivo. Unfortunately, it is difficult to precisely track the rate of in vivo biodegradation of synthetic polymers.

## 3. Materials for Producing Scaffoldless CEC Sheets

A promising approach involves cultivating cells in a setting that encourages more interaction between cells, producing a single cell layer with desired implantation and biological function characteristics. Biomaterials have emerged as a potential solution for promoting interaction between cells and the detachment of a cohesive cell layer. Specifically, stimuli-responsive polymers can alter their molecular structures or physicochemical properties based on environmental cues, enabling the scaffoldless production of CEC sheets.

### 3.1. Biologically Derived Materials

Extracellular matrix (ECM) proteins are of particular significance for the attachment and proliferation of CECs in culture, given their biological origin. Collagens, fibrinogen, and laminins are among the ECM proteins that are most commonly and thoroughly examined. Among them, collagen is a typical natural material that can be used to culture CECs, which form a continuous monolayer on top and are subsequently detached for transplantation [22]. Combining collagen with other polymers, such as diethylene glycol methacrylate, has tuned the temperature for detachment to ~32 °C [23]. The consequent cell layers can be implanted in denuded rabbit corneas, gradually enhancing clarity compared to control groups. In another circumstance, a temperature drop of up to 20 °C could occur without adversely affecting the detachment of a cell layer [24].

### 3.2. Synthetic Materials

**Figure 2:**
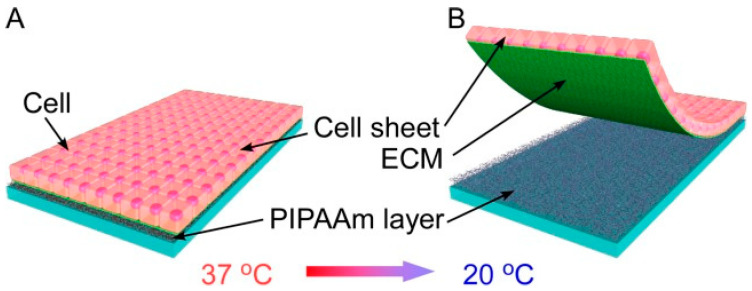
The schematic illustration of the cell sheet on a PIPAAm-modified surface: (**A**) At 37 °C, cells can adhere to the dehydrated PIPAAm layer, and (**B**) at 20 °C, cells can detach from the hydrated PIPAAm layer with ECM, resulting in an intact and contiguous cell sheet [25]. Reprinted with permission from Ref. [25]. Copyright 2014, Wiley Periodicals, Inc.

Indeed, numerous polymers responsive to various stimuli are utilized as biomaterials. Among them, the temperature-responsive polymer poly(N-isopropyl acrylamide) (PNIPAm) has emerged as one of the most promising synthetic polymers for producing cell sheets. It has already been successfully employed in clinical settings for expanding corneal epithelial cells [25]. The precise control of temperature enables the controlled adhesion at an optimal temperature of 37 °C and detachment at a reduced temperature of 20 °C of the cells from PNIPAm, as depicted in Figure 2. Numerous studies have demonstrated the capacity of PNIPAm to support key structures of CECs, including the Na^+^/K^+^-ATPase pump, and maintain cellular morphology with microvilli and cellular interconnections [26]. To evaluate cell functioning following culture on PNIPAm, rabbit models have been used. Soh et al. [27] synthesized a triblock copolymer of PNIPAm-poly[(R)-3-hydroxybutyrate] (PHB)-PNIPAm, achieving the construction of bovine corneal endothelial cell sheets. This terpolymer material could coat the substrate, enhance the proliferation rate of bovine corneal endothelial cells, and effectively separate intact cell sheets without affecting cell viability through temperature changes.

These thermoresponsive substrates can be cultivated without serum as a crucial step toward therapeutic application [28]. In conjunction with various ECM coatings, thermo-responsive polymers facilitated cellular adhesion.

Despite the extensive research on stimuli-responsive polymers, they still require further investigation for their function in corneal endothelium layer transplants and the impact of temperature change on CECs’ bioactivity. Moreover, only using cell sheets limits the surgical choices available due to the sheets’ fragility, necessitating more invasive procedures. To address this issue, using supporting materials as scaffolds for CEC cultivation and transplantation has been the subject of various investigations [15].

## 4. Materials for Producing Scaffolds of CECs

The cultivation of CECs is a delicate and challenging process to manage. Consequently, using substrates provides mechanical stability while performing the transplantation of ex vivo-designed human corneal endothelial sheets. Furthermore, they can also produce the favorable milieu required for the activity of cells. The substrate’s biological, mechanical, chemical, and physiological characteristics should ideally resemble DM’s.

### 4.1. Substrates

Two distinct strategies can be distinguished for substrate development: one involves using natural ECM-derived membranes (such as lens capsules, fish scales, or amniotic membranes), while the other involves creating entirely new scaffolds using natural or synthetic polymers. The advantages and limitations of substrate materials for CEC scaffolds are summarized in Table 1.

#### 4.1.1. Natural Substrates

The utilization of an amniotic membrane (AM) has a significant advantage since it is a biologically derived membrane that is natural, inert, and non-cytotoxic. Since it has demonstrated biocompatibility in ocular applications, using AM decreases the risk of potential graft rejection. Studies conducted in cats [29] and rabbits [30] have demonstrated favorable outcomes in preserving a corneal endothelial layer with a proper thickness, which is essential for the refraction of incoming light. While AM has advantages such as biocompatibility and preservation of corneal endothelial layer thickness, it also has some drawbacks. These include its semi-opaque nature, the scarcity of donor banks, and the possibility of contamination and the spread of infectious illnesses. Its application in ocular endothelial tissue engineering has also been hampered by biological heterogeneity among donor tissues, inadequate transparency, and unpredictably high deterioration rates. Additionally, it has been investigated whether corneas devoid of endothelium cells could serve as substrates for CECs [31]. They are suitable without significant alteration, given that they offer the appropriate form, mechanical backing, and transparency. ZO-1, Na^+^K^+^ATPase, and connexin were expressed in human corneal endothelial grafts using CECs cultivated in vitro on decellularized human corneal stroma [32]. Other membranes or films derived from the ECM of natural sources, such as lens capsules, have also been studied in corneal endothelium grafts, but their use does not decrease the dependence on donors [33].

Natural polymers are organic compounds from diverse biological sources, including plants, animals, and microorganisms. This class of polymers is characterized by macromolecules consisting of proteins or polysaccharides such as collagen, alginate, starch, and chitosan. They appeal to biomedical applications due to their biocompatibility, biodegradability, and negligible cytotoxicity. Additionally, they replicate the extracellular matrix (ECM), which is a sought-after trait in the biomedical domain. Type I collagen, constituting 25–30% of all proteins in the human body, is a vital structural component of connective tissues, including but not limited to muscle, teeth, bone, and skin. Vazquez et al. [34] developed an artificial corneal endothelial graft using purified human type I collagen membranes and confluent human and rabbit corneal endothelial cells expressing distinctive corneal endothelium markers. Following this, the artificial lamellar endothelium was transplanted into a rabbit model with endothelial dysfunction. Remarkably, full restoration of corneal transparency and thickness was achieved within six weeks.

A composite material comprising hydroxyethyl chitosan, gelatin, and chondroitin sulfate was synthesized [35]. Regarding cultivating corneal endothelial cells on the substrate, the outcomes were positive, as evidenced by a noteworthy increase in cell proliferation on the fourth day. Moreover, membrane permeability and cell organization were comparable to the native human cornea. The construct was subsequently transplanted into the anterior chamber of rabbit models, where it maintained its transparency and structure for three weeks. Although inflammation was initially observed at the corneal-iris interface, endothelial cell dystrophy was not mentioned after three weeks, which is a crucial consideration since endothelial cell loss may result from inflammation. While these outcomes are encouraging, this approach is still in the exploratory phase and necessitates further research to determine its suitability for human use. In another investigation, agarose (AG) underwent modification with various attachment signals, including arginylglycylaspartic acid (RGD), lysine, polylysine, and fish-derived gelatin. Samples with different conjugation ratios were produced. All resulting products formed bulk hydrogels, which collapsed into ultrathin membranes under controlled conditions. The hydrated AG membranes hold great potential as scaffolds for corneal endothelial cell culture during endothelial keratoplasty [36].

This natural material class has several drawbacks. Natural polymers exhibit batch-to-batch differences due to natural variability, resulting in inconsistent properties among polymers obtained from the same source. Furthermore, materials obtained from natural sources may cause immune reactions or transmit pathogens. Although naturally sourced polymers are often inexpensive to produce, their manufacture relies on environmental factors that are challenging to regulate, which makes the process relatively sluggish. Additionally, certain biomedical manufacturing applications cannot be supported by natural polymers because of their poor thermal and mechanical properties.

#### 4.1.2. Synthetic Substrates

The examination of synthetic polymers as an alternative for biomedical applications has been undertaken to address these concerns. Natural polymers have batch-to-batch discrepancies, whereas synthetic polymer synthesis is incredibly repeatable, allowing for predictable chemical and physical characteristics. In contrast to natural polymers, higher volumes can typically be produced. Additionally, it is simple to change the characteristics of synthetic polymers to receive the expected outcomes.

Poly (DL-lactide-co-glycolide), a composite of polylactic acid (PLA) and glycolic acid, has been demonstrated to have a positive impact on CECs [37]. Interestingly, the biocompatibility of the membranes decreased with an increase in the amount of PLA present in the samples, likely owing to a faster degradation rate that caused the culture medium’s pH to become more acidic, ultimately leading to decreased cell viability.

To assess their potential impact on clinical demand and scientific relevance, various hydrophobic biomaterials, including tissue culture polystyrene, poly(ethylene-co-vinyl alcohol), polyvinylidene fluoride (PVDF), and polyvinyl alcohol, were tested in the bovine corneal endothelial cells (BCECs) culture system [38]. When cultured on PVDF, BCECs exhibited well-developed expressions of gap junction connexin-43, differentiation marker N-cadherin, and tight junction ZO-1, closely resembling physiological phenotypes. This indicates that biomaterial properties impact the adhesion and proliferation of BCECs and significantly influence the transformation and phenotype expression of these cells.

Another highly promising synthetic polymer of exceptional transparency is polyethylene glycol. It can be used to produce thin membranes with a tensile strength comparable to that of human corneal tissue, a thickness of 50 μm, and a transparency exceeding 98% [15]. Moreover, the multiplication of CECs was aided by these substrates, resulting in a fully confluent layer after seven days, enabling its implantation in a sheep corneal model. While the outcomes were expected, additional research is necessary to further evaluate the biological activity of biodegradable systems to enhance cell–material interaction.

Kennedy et al. [39] suggested employing a synthetic peptide hydrogel comprising poly-ε-lysine (pεK) cross-linked with octanedioic acid as a substrate for CEC expansion using both human CEC lines (HCEC-12) and primary porcine endothelial cells. In contrast to porcine CECs (pCECs), which needed the functionalization of the pεK hydrogel with a synthetic cell-binding peptide in order to adhere, the HCEC-12 adhered and established confluent monolayers on pεK hydrogels after seven days. When ZO-1 and Na^+^/K^+^-ATPase were expressed at the end of the fifth week, this improved the adhesion and development of pCECs, indicating the formation of a functional corneal endothelial layer. Synthetic biomaterials have the advantage of being customizable, as their inherent characteristics can be precisely manipulated to create a customized material. Moreover, the biomaterial in question possesses several free amine sites that can be functionalized with synthetic peptides, further increasing its customizability. Therefore, this synthetic peptide hydrogel demonstrates significant potential as a suitable scaffold for creating a tissue-engineered corneal endothelial graft.

In recent years, there has been a growing fascination with producing tissue-engineered scaffolds through electrospinning. Kruse et al. [19] conducted experiments to assess the feasibility of cultivating human CECs on electro-spun scaffolds made of three distinct synthetic polymers: poly(methyl-methacrylate) (PMMA), poly(lactic-co-glycolic acid) (PLGA), and polycaprolactone (PCL). The results indicated that PMMA had substantial cytotoxicity, whereas electro-spun scaffolds made of PLGA and PCL showed comparable biocompatibility, but only PLGA sustained the morphology of CECs. The human CECs, on the other hand, were able to grow on the surface while preserving their morphology because the PLGA fibers, which were the thinnest, were tightly packed. However, electrostatic spinning has limitations in precisely controlling the network structure. Electro-spun fibers are so dense that cells inoculated on the fiber surface cannot migrate to the interior of the fiber. Dense electro-spun fibers may also reduce the light transmission in a scaffold, making it unsuitable as a replacement material for corneal tissue.

When using synthetic polymers for biomedical applications, caution must be taken as they are not made from natural materials and might not even be biocompatible. Additionally, inflammatory responses have been detected during in vivo experiments with some synthetic polymeric materials in animals. Vijayasekaran et al. [40] implanted poly(hydroxyethyl methacrylate) (PHEMA) porous hydrogel material into rabbit corneas and observed a significant percentage of invasive inflammatory cells after 12 weeks. Gao et al. [41] synthesized a hydroxypropyl chitosan composite membrane, and an acute inflammatory response was found in the skeletal muscle of rats after two weeks of material implantation. Despite the fact that synthetic polymers have demonstrated promising outcomes, further in vivo studies are necessary to validate their safety and effectiveness.

**Table 1:** Summary of advantages and limitations of substrate materials for CEC scaffolds.

Category	Type	Advantages	Limitation	Ref.
natural substrates	amniotic membrane	biocompatibility in ocular applications	semi-opaque; scarcity from a donor bank; biological variability; unpredictable degradation rates	[29,30]
	decellularized human cornea	mechanical support, and transparency	donor dependency	[31,32]
	collagen	biocompatibility and biodegradability; mimic the ECM	transparency not optimal	[34]
	hydroxyethyl chitosan, gelatin, and chondroitin sulfate	promote cell proliferation	inflammation	[35]
	agarose	ultrathin; strong; cell-adhesive	no use with human CECs	[36]
synthetic substrates	poly (DL-lactide-co-glycolide)	a positive impact on CECs	a faster degradation rate resulting in a more acidic pH in the culture media; no in vivo studies	[37]
	polyvinylidene fluoride	BCECs exhibited well-developed expressions of specific markers	no in vivo studies	[38]
	polyethylene glycol	high tensile strength and transparency; facilitate the proliferation of CECs	need further evaluation of the biological activity of the biodegradable system	[15]
	poly-ε-lysine	enhanced adhesion and growth of pCECs	no in vivo studies	[39]
	poly(methyl-methacrylate)	inexpensive; modifiability of transparency	cytotoxicity; lowest viscosity	[19]

### 4.2. Modifications of the Substrates

#### 4.2.1. Changes in the Composition

It is common practice to combine or modify synthetic polymers with natural polymers in order to adjust their characteristics toward certain targets and increase their biocompatibility. A hybrid or copolymer can be created by chemically combining natural and synthetic components.

Various blends of artificial and natural polymers, including mixes of glycerol and silk fibroin, have been implemented. While glycerol presents an impressive level of transparency, it lacks the required mechanical stability and has limited biological interactions. Similarly, in isolation, silk fibroin does not possess enough transparency, despite having excellent biocompatibility and mechanical characteristics [42]. Nonetheless, during preparation, cracks could form. It has been demonstrated that the addition of glycerol reduces these cracks. As a result, their interaction has produced a rather homogeneous structure with a thinner layer and a rougher surface. The outcomes indicate that CECs exhibit better proliferation on surfaces with intermediate roughness when combined with 1% glycerol in silk fibroin. Incorporating aligned nanofibers made of polylactic acid and glycolic acid into the sheets of compressed collagen can also enhance its mechanical characteristics [43]. The in vitro culture of CECs manifested excellent behavior, displaying appropriate morphology and adequate phenotype expression.

Polylysine, when used as a substrate, can be altered with artificial peptides such as RGD to boost CECs’ activity. Moreover, combining it with proteins derived from natural sources improves cell adhesion and functionality [39]. In a similar vein, the silk fibroin surface has been coated with beta-carotenoids to boost proliferation and enhance phenotypic response. Beta-carotenoids are acknowledged for their antioxidant properties, which protect patients with eye diseases. The outcomes demonstrate an improvement compared to the unmodified silk film, primarily in terms of CEC marker expression and proliferation [44].

Gelatin is a natural biological material obtained by the hydrolysis of collagen, one of the main components of human tissues. It contains RGD sequences, which provide a good substrate for cell attachment. Gelatin undergoes a sol–gel transition at about 30 degrees. And the cost of gelatin is much lower as compared to collagen. Gelatin hydrogel has been used as a CEC carrier, but its excessively fast degradation rate and low mechanical properties make it a poor match for the performance of DM [45]. Therefore, researchers tried to obtain cross-linked gelatin materials by means of methacrylic anhydride modification to enhance their mechanical properties. Research has indicated that gelatin methacrylate (GelMA) has the potential to serve as a biomaterial for ocular tissue engineering. Rizwan et al. [46] have modified GelMA for use as a scaffold for a tissue-engineered CEC monolayer. They devised a sequential hybrid cross-linking process followed by UV cross-linking to create an advanced material called GelMA+. Compared to ordinary GelMA, it showed an eight-fold increase in mechanical strength and slower in vitro and in vivo degradation rates. Additionally, they were able to generate hydrogel pattern topographical cues with a resolution of 1 μm or less, which enhanced cell growth and viability. CEC monolayers grown on GelMA+ scaffolds displayed ZO-1 expression, increased cell density, and homogeneous cell size as opposed to GelMA, indicating the superiority of the functional monolayers. Van et al. [47] reported the production of artificial Descemet membranes using a blend of biodegradable amorphous polyester (poly (d,l-lactic acid)) and cross-linkable gelatins. The fabrication process involved multi-step spin-coating, including a sacrificial layer to permit easy membrane detachment after production. This results in very translucent (>90%), ultrathin (<1 μm), and semi-permeable membranes with significant physiological potential. The membranes encouraged corneal endothelial cells’ correct phenotypic and distinctive morphology, and they showed comparable cell proliferation rates to the positive control. However, the production of cross-linked gelatin stents is relatively lengthy and expensive. Thus, further in vivo experiments are required.

Both polycaprolactone (PCL), a biodegradable polyester, and chitosan, a biomimetic polysaccharide, are recognized as biomaterials by the Food and Drug Administration. Wang et al. [39] have successfully created blended membranes by combining chitosan and PCL. Chitosan is weak in strength, but PCL is strong and encourages the formation of ECM and cell adhesion. The mixed membrane was found to be advantageous for the expansion of CECs, maintaining their phenotype in vitro. After 14 days of incubation, CECs cultured on a 25% PCL and 75% chitosan blended membrane expressed the tight-junction protein ZO-1, the Na^+^/K^+^ ATPase, and the gap junction protein connexin-43, confirming their functional features. The same team demonstrated in recent work that CECs cultivated on hybrid membranes may create their own ECM containing collagen IV, which can be found in DM [48]. Additionally, chitosan and polyethylene glycol have been mixed to create ultrathin hydrogel membranes that exhibit excellent transparency and mechanical properties on par with or better than those of human corneal tissue. These films were successful in ex vivo surgical tests on ovine eyes and supported the attachment and growth of sheep CECs [49].

CECs were successfully enclosed in chitosan, hydroxypropyl chitosan, and sodium alginate before being applied to DM [50]. Remarkably, the CECs remained viable and maintained their normal morphology. This presents a novel prospect for corneal endothelium restoration via the in-situ formation of hydrogels. An injectable system of oxidized dextran hydrogels cross-linked with adipic acid dihydrazide was employed. Notably, by adjusting the level of dextran oxidation and the ratio of the two constituents, the gelation time and degradation properties could be regulated. Findings indicated that cell adhesion and proliferation were feasible, despite the fact that lightly oxidized dextran achieved confluence in just 24 h of culture [51]. Additionally, the released products from the dextrans exhibited no cytotoxicity in indirect cultures, demonstrating exceptional biocompatibility.

Furthermore, functional nanomaterials can serve as reinforcement to improve the performance of the corneal endothelial scaffold. Carbon quantum dots (CQD) were reported to have applications in ocular nanomedicine [52]. It was shown that positively charged CQDs derived from glucosamine hydrochloride and spermidine (CQD-S) could effectively enhance the permeability of glucose and could be used as a permeation enhancer for corneal endothelial therapy. Gold nanoparticles (GNPs) and superparamagnetic iron oxide nanoparticles (SPIONs) have been utilized as cellular tracers in ocular therapy. The application of nanoparticles helps track the fate of ocular transplanted cells.

#### 4.2.2. Regulation of Topography

**Figure 3:**
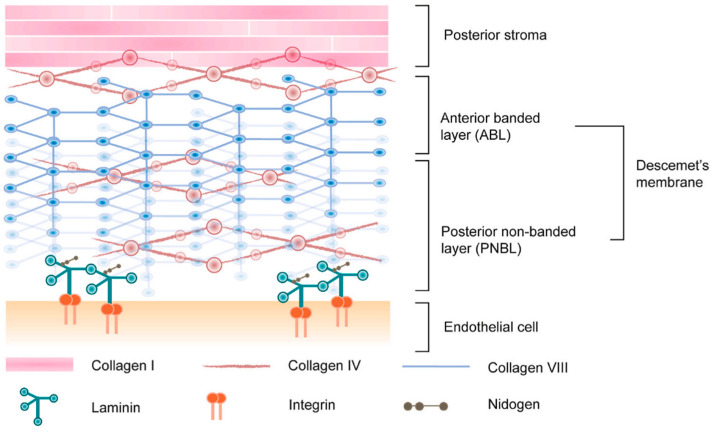
Schematic of the ECM structure of Descemet’s membrane [53]. Reprinted with permission from Ref. [53]. Copyright 2021, Elsevier Ltd.

The composition is significant, and the matrix’s topographic features are even more critical. The principal constituents of an ECM consist of collagen IV and collagen VIII, both of which are synthesized by CECs. Collagen VIII can generate a distinctive hexagonal arrangement when assembled and is the primary ECM framework within DM. The basal aspect of CECs exhibits dendritic extensions that interconnect with neighboring cells and adhere to the posterior surface of DM. Integrin binding sites establish a connection between the cell surface and the ECM. The extracellular domains of integrins attach to matrix molecules such as laminin, nidogen, fibronectin, and collagen, as schematically shown in Figure 3 [53]. This evidence supports the idea that the corneal endothelium and the ECM have a strong structural connection. Engineering a similar construct can provide a suitable ECM environment to potentially support the growth of CECs.

Recent research has demonstrated that adding surface topography helps modify materials’ surface qualities, including hydrophilicity, surface energy, and cell interactions, without altering the bulk features of the substrate material [54,55,56,57]. Topographical signals pertain to the 3D arrangement of the ECM surface, encompassing its shape, geometry, size, and organization. These characteristics are influenced by the alignment and texture of the ECM, which include the designs on fibrils, pores, and pits. The topography of the ECM exerts a significant influence on cell morphology, migration, and differentiation, thereby playing a crucial role in maintaining tissue homeostasis.

Various methods for patterning materials have been developed based on specific requirements and designs. These include direct laser interference patterning [58], template-based surface nanopatterning [59], nanoimprinting [60], and soft lithography [61]. The characteristics of the materials and the desired pattern influence the process selection.

Over the last few decades, numerous studies have demonstrated that surface topography can alter the wetting state of a material in addition to its inherent hydrophobic or hydrophilic properties. Dai et al. [62] have demonstrated that the height and width of pillar constructions can influence the interaction between droplets and substrates. Specifically, the height of pillars with a width of 2.82 nm can be increased to 3.76 nm to alter the wetting state of the surface from the Wenzel state to the Cassie–Baxter state when the water contact angle on a smooth surface surpasses 93.13°. However, when the water contact angle on the smooth substrate is less than 85.1°, the effect of pillar sizes on the wetting condition is absent. Studies have demonstrated that the hydrophobicity of hydrogels can modulate cellular behaviors, including adhesion and migration [63].

Proteins, a vital constituent in human body fluids, can be rapidly adsorbed onto the outer layer of biomaterials upon exposure, triggering cell responses [64]. However, in some fields similar to contact lens studies, the adsorption of tear film components, such as proteins and lipids, onto the lens material can cause discomfort or even severe eye symptoms [65]. Thus, developing biomaterials capable of resisting non-specific protein adsorption is crucial for anti-fouling surfaces and other applications with clear chemical structures or desired bioactivities. Recent research has demonstrated that adding surface topography to hydrogels can modify protein adsorption. To further support this hypothesis, experiments were conducted to investigate the adhesion of proteins onto surfaces of patterned hydrogels, such as bovine serum albumin (BSA), bovine fibronectin (FN), and bovine vitronectin (VN). Results showed that either bovine FN or bovine VN preferred to adhere to the groove walls on the surfaces with line patterns [66,67].

Surface topographies can influence bacterial motility by interacting with bacterial appendages such as flagella and pili. These topographies can be utilized to achieve antibacterial functions, such as creating bactericidal or anti-adhesion surfaces. Anti-adhesion surfaces are designed to prevent bacterial cells from adhering to the top by offering an undesirable topology. Studies have shown that smaller topographies are more effective in reducing bacterial adhesion than larger structures [68]. Corneal endothelial scaffolds with antimicrobial properties help to preserve the normal ocular microbiota during cell culture, avoiding chronic inflammation and biofilm formation [69].

Micro- and nano-topography have been developed on substrates and implants to mimic the natural extracellular environment, with the goal of modulating cell behaviors in vitro and in vivo. Previous studies have demonstrated that CECs can detect biomechanical variations in their environment and, in response, control the formation of the extracellular matrix (ECM) throughout physiological and pathological processes [70]. A mechanotransduction signaling mechanism has been used to characterize the dynamic, reciprocal interaction between CECs and their surroundings. During this process, biophysical cues from the ECM can be transformed into intracellular biochemical signals that trigger responses from cells. Topographical cues can affect cell cytoskeletal configuration through the JNK-ERK1/2 and PI3K pathways [53]. The mean cell area of human CECs ranges from 332.3 ± 46.3 μm^2^ to 390.59 ± 149.94 μm^2^, enabling cells to perceive topographical features ranging in size from nanometers to micrometers, such as those lying on fibrils [71]. However, a single cell may not detect topographical features larger than 2 μm.

**Figure 4:**
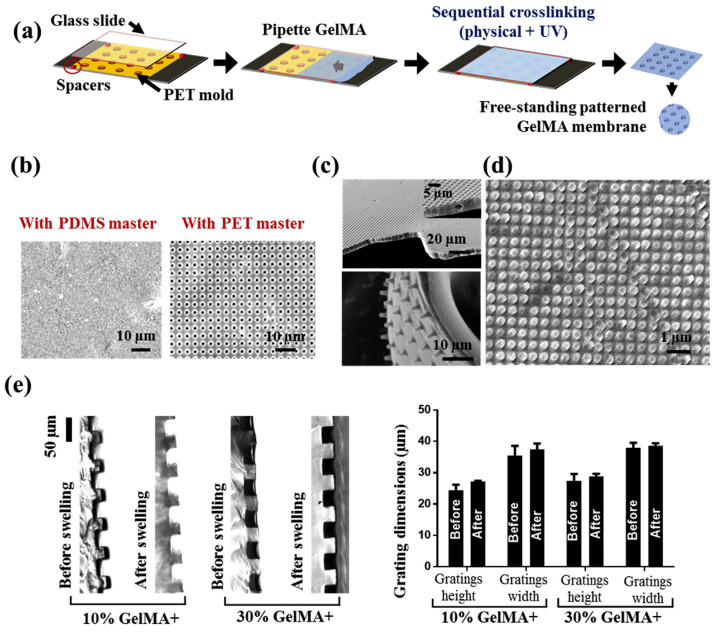
High-resolution UV patterning of 30% GelMA+ films: (**a**) schematic diagram of the micro and nano-molding method by using polyethylene terephthalate (PET) stamps to pattern hydrogel films; (**b**) effect of stamp mold material (polydimethylsiloxane vs. PET) on the replication of high resolution for 1-μm features; (**c**) fabrication of high aspect ratio pillars and wells on GelMA+ thin films; (**d**) fabrication of nanopatterns (250 nm pillars) by using PET stamp-based nano-molding; and (**e**) effect of hydration on the dimensions of surface topography of GelMA and GelMA+ hydrogels as measured in terms of the change in dimensions of grating height and width before and after swelling. (*n* = 4) [46]. Reprinted with permission from Ref. [46]. Copyright 2017, Elsevier Ltd.

The production process of materials can create patterned structures at the micro and nano levels, providing a fascinating method to govern the fate of CECs. For example, patterned structures were produced using gelatin methacrylate, as shown in Figure 4. It was discovered that the gratings’ presence, which varied in height and width, had a direct impact on CEC activities. In particular, it was discovered that gratings with 1 μm square and hexagonal pillars proved to be optimal [46]. Furthermore, poly (glycerol sebacate) (PGS)-PCL blends were electro-spun into nanofibrous structures [72]. The CECs cultured on these nanofibrous films created monolayers with hexagonal topologies. Notably, an increase in the PGS/PCL ratios resulted in improved organization and functionality of the CECs. The studies suggest that scaffold materials with specific surface topographies are beneficial for promoting CECs’ adhesion, growth, and differentiation. This may be related to the effects of surface topography on material hydrophobicity, protein adsorption properties, microbial adhesion properties, and cellular response characteristics.

### 4.3. Scaffolds with Drug Delivery System

Cells can communicate with each other by releasing signaling molecules. These molecules can cause recipient cells’ morphologies to alter, changing those cells’ phenotypes and eventually triggering regeneration pathways. Most of the materials employed in CEC tissue engineering are made of water-soluble polymers. This means that signaling molecules can be trapped inside these matrices to allow continuous release over time and mimic ideal phenotypes and regeneration pathways. Gelatin has a remarkable propensity to absorb water and, when coupled with heparin, can be finely tuned to produce membranes that encapsulate growth factors such as a basic fibroblast growth factor. This encapsulation has been demonstrated to boost cells’ ability to proliferate. These membranes have the capacity to continuously release growth factors. It is significant to point out that the cells grown on these membranes have shown suitable CEC-specific surface indicators and enough stiffness for manipulation and implantation [73]. These findings provide evidence for using drug-eluting biomaterials for corneal implantation as an alternative to placing cell sheets or scaffolds with cells directly into the cornea.

In addition to growth factors, drug molecules can also be released from such materials. For example, dorzolamide hydrochloride was coated on the surface of PCL-based materials and was shown to be released once implanted in rabbit eyes, causing the intraocular pressure to drop [74]. Additionally, these materials have demonstrated biocompatibility with CECs. However, no understanding of the cellular markers has been provided. To achieve near-zero-order kinetics instead of burst release, membrane optimization is required. This might be achieved by integrating medications within the scaffolds instead of relying solely on coatings. A more advanced system involves the preparation of microspheres from biodegradable PLGA containing the ROCK inhibitor Y-27632 and utilizing gelatin in a double emulsion. This was injected into the anterior chamber of rabbit eyes [75]. The use of ROCK inhibitors as a clinical treatment has been proposed, but long-term immunosuppressive treatments are still required to prevent any immune response. Encapsulation of the ROCK inhibitor within microspheres has enhanced CEC proliferation in vitro, with release rates controlled by adjusting the lactic-to-glycolic ratio. While positive results have been obtained in vitro, the release of the drug was observed to be complete within 10 days, limiting the potential long-term outcomes of this approach. Therefore, alternative encapsulation systems that permit a more prolonged release must be explored, along with methods to boost the long-term administration of medications.

## 5. Challenges and Prospects of Scaffolds for Corneal Endothelium Tissue Engineering

Many polymeric materials have been applied to corneal endothelial tissue engineering research today, and some progress has been made. However, the following challenges still need to be addressed:

(i) It is important to note that meeting all requirements for a tissue-engineered corneal endothelial graft remains a challenging task. It should be acknowledged that the important properties of a graft are interdependent. For instance, altering the thickness of a scaffold can impact its permeability and mechanical properties. The increase in mechanical strength may be accompanied by a decrease in water content, thus affecting cytocompatibility. Researchers need to balance as many performances as possible through exquisite scaffold design.
(ii) Although many designs of scaffold topography have been shown to promote endothelial cell growth, the mechanism of this effect is unclear. The functional implementation of bioengineered CECs is not yet ideal due to a limited understanding of the molecular mechanisms of endothelial cell proliferation and the related inter- and intra-signaling pathways that maintain the dynamic balance of corneal endothelial tissue.
(iii) For biodegradable scaffolds, the match between their degradation rate and the regeneration rate of DM needs to be further investigated. However, there are few reports on the regeneration of human DM in vivo, making the study of degradation rates of scaffolds challenging.

Membranes constructed from multiple components exhibit the most promising potential to meet all the requirements. Specifically, they enable the combination of properties that cannot be achieved using a single material. Furthermore, combining several different methods could be helpful. For instance, the structural layer may be made using spin coating, which enables the manufacture of the thinnest films. In contrast, the cell-interactive layer could be made by solvent casting, and topographical cues could be provided to the cells by a textured mold. Combining several methods would guarantee the development of a thin, translucent, strong, and permeable scaffold that permits the best possible cell response. As a result, we suggest that future research concentrate on blending different materials to create a scaffold that meets as many requirements as possible.

It is necessary to conduct further research to better understand how surface topography affects cells, using advanced methods such as two-photon polymerization and nano-thermoforming that may be useful in creating scaffolds with intricate topographies. In-depth studies on the molecular mechanisms of endothelial cell proliferation and the mechanisms of topological morphology are needed to guide the rational design of scaffold materials. Once clinical trials of corneal tissue engineering begin, valuable data on natural DM regeneration will be collected, and research on biodegradable corneal endothelial scaffolds will be further advanced.

## 6. Conclusions

Significant advancements have been achieved in the realm of tissue engineering for corneal endothelial grafts. Utilizing bioengineered scaffold materials offers several advantages over biologically sourced alternatives. For instance, it allows for more accurate control over variability and tuning based on desired properties, enhancing reproducibility and enabling mass production with batch-to-batch consistency. In light of the aforementioned characteristics, it can be summarized that a substrate should be produced with sufficient transparency, mechanical stiffness, the permeability of nutrients, biodegradability/biostability, strong attachment to the posterior surface of a cornea, and biocompatibility. It must provide a microenvironment for cell proliferation and adhesion, stimulating collagen secretion. In this regard, employing additional processing steps or more complicated scaffold designs holds considerable promise. The design of surface topography with advanced techniques may be beneficial in producing scaffolds with optimized potential to promote cell growth once the mechanism is clear. Finally, both optimizing human CEC culture techniques and identifying specific markers for human CECs are valuable. We anticipate future research progress from the in vitro stage to animal studies and potentially early human trials.

## Data Availability

No new data were created or analyzed in this study. Data sharing does not apply to this article.
